# Whole genome analysis of *Bacillus velezensis* 160, biological control agent of corn head smut

**DOI:** 10.1128/spectrum.03264-23

**Published:** 2024-02-16

**Authors:** Yared González-León, Esaú De la Vega-Camarillo, Rocío Ramírez-Vargas, Miguel Angel Anducho-Reyes, Yuridia Mercado-Flores

**Affiliations:** 1 Universidad Politécnica de Pachuca, Zempoala, Hidalgo, Mexico; 2 Escuela Nacional de Ciencias Biológicas, Instituto Politécnico Nacional, Mexico City, Mexico; Institut National de Santé Publique du Québec, Sainte-Anne-de-Bellevue, Canada

**Keywords:** *Sporisorium reilianum*, rhizobacteria, antifungals, secondary metabolites, phytopathogens, corn production

## Abstract

Corn head smut is a disease caused by the fungus *Sporisorium reilianum*. This phytosanitary problem has existed for several decades in the Mezquital Valley, an important corn-producing area in central Mexico. To combat the problem, a strain identified as *Bacillus subtilis* 160 was applied in the field, where it decreased disease incidence and increased crop productivity. In this study, the sequencing and analysis of the whole genome sequence of this strain were carried out to identify its genetic determinants for the production of antimicrobials. The *B. subtilis* 160 strain was found to be *Bacillus velezensis*. Its genome has a size of 4,297,348 bp, a GC content of 45.8%, and 4,174 coding sequences. Comparative analysis with the genomes of four other *B. velezensis* strains showed that they share 2,804 genes and clusters for the production of difficidin, bacillibactin, bacilysin, macrolantin, bacillaene, fengycin, butirosin A, locillomycin, and surfactin. For the latter metabolite, unlike the other strains that have only one cluster, *B. velezensis* 160 has three. A cluster for synthesizing laterocidine, an antimicrobial reported only in *Brevibacillus laterosporus*, was also identified.

**IMPORTANCE:**

In this study, we performed sequencing and analysis of the complete genome of the strain initially identified as *Bacillus subtilis* 160 as part of its characterization. This bacterium has shown its ability to control corn head smut in the field, a disease caused by the basidiomycete fungus *Sporisorium reilianum*. Analyzing the complete genome sequence not only provides a more precise taxonomic identification but also sheds light on the genetic potential of this bacterium, especially regarding mechanisms that allow it to exert biological control. Employing molecular and bioinformatics tools in studying the genomes of agriculturally significant microorganisms offers insights into the development of biofungicides and bioinoculants. These innovations aim to enhance plant growth and pave the way for strategies that boost crop productivity.

## INTRODUCTION

Agriculture is one of the main anthropogenic activities due to its direct relation to food provision; so, it has both economic and social impacts. Among the critical problems that affect agricultural production and put food security at risk is the excessive use and mismanagement of agrochemicals that can have harmful effects that lead to the degradation of agroecosystems (1–3).

Corn is, undoubtedly, a major cereal worldwide. Its production is intended, mainly, for human and animal consumption, though it also has applications in various kinds of industries (4). In Mexico, vast expanses of agricultural land are utilized to cultivate corn (5). The Mezquital Valley, in the state of Hidalgo, is the region with the highest corn production. There, irrigation is conducted with sewage water, sowing is done by using traditional methods, and there is widespread utilization of high-yielding hybrids that necessitate high inputs of agrochemicals (6).

The phytosanitary problems in this area include corn head smut, a disease with worldwide distribution that in Mexico is concentrated in the central states. The etiological agent is the phytopathogenic fungus *Sporisorium reilianum*, which causes enormous losses in crop yields and, therefore, reduces economic gains (7). The infection occurs during seed germination or in the first 15 days of seedling development but signs and symptoms are manifested until flowering, when carbonaceous masses made up of teliospores are observed in the spikes and ears. These fungal structures are the primary means of spreading this disease, as they can carried through the soil by wind, rain, and agricultural equipment, where they remain viable for several years. Under adequate conditions, the teliospores germinate, producing four basidiospores with distinct sexual compatibility. The ones that are compatible merge to form the mycelial infectious phase that comes into contact with the young tissues of the plants, where it penetrates and colonizes to establish a systemic infection (8–10).

Head smut control normally consists of using tolerant hybrids and chemical fungicides; however, these strategies favor the selection of resistant strains of the phytopathogen (9, 11, 12). In this case, biological control is an option. This approach is defined as the use of living organisms to reduce the population density of a pathogenic organism or pest to make it less harmful to crops (13). The most widely used biocontrol agents include several members of the genus *Bacillus*, a group of bacteria with a broad distribution in nature. They are Gram-positive, rod-shaped, producers of endospores, and aerobic and facultative anaerobes. Some species have been identified as plant growth-promoting rhizobacteria (PGPR) due to their ability to produce phytohormones, fix nitrogen, and solubilize phosphate. In addition, they are essential for controlling pests and diseases by producing substances with insecticidal and antimicrobial action, as well as stimulating the plant’s own defense mechanisms (14–16).

Given the importance of the genus *Bacillus* in agriculture, in recent years, the sequencing of the genomes of several strains has been a fundamental part of the characterization process and has contributed to identifying, at the genetic level, the mechanisms in these microorganisms that benefit healthy plant growth (17–23). Petatán-Sagahón et al. (24) isolated numerous bacteria from the maize rhizosphere collected in crops in the Mezquital Valley and demonstrated their ability to inhibit the development of the phytopathogenic fungi *Stenocarpella maydis* and *Stenocarpella macrospora*. They found that strain 160 exhibited a high antifungal effect. This strain was identified by amplification and sequence analysis of the 16s rDNA gene. The phylogenetic tree obtained made it possible to establish its association with the *Bacillus subtilis* group. This bacterium was evaluated to determine its effect on controlling corn head smut, showing that its application decreased disease incidence and increased crop productivity (25).

### Objective of this study

Using new sequencing and bioinformatics tools, this study aimed to analyze the genome of the strain identified as *B. subtilis* 160 to clarify its taxonomic position, understand its relation to other strains, and identify the genetic determinants that enable it to exert biological control.

## RESULTS

### Phylogenomic analysis

**Fig 1:**
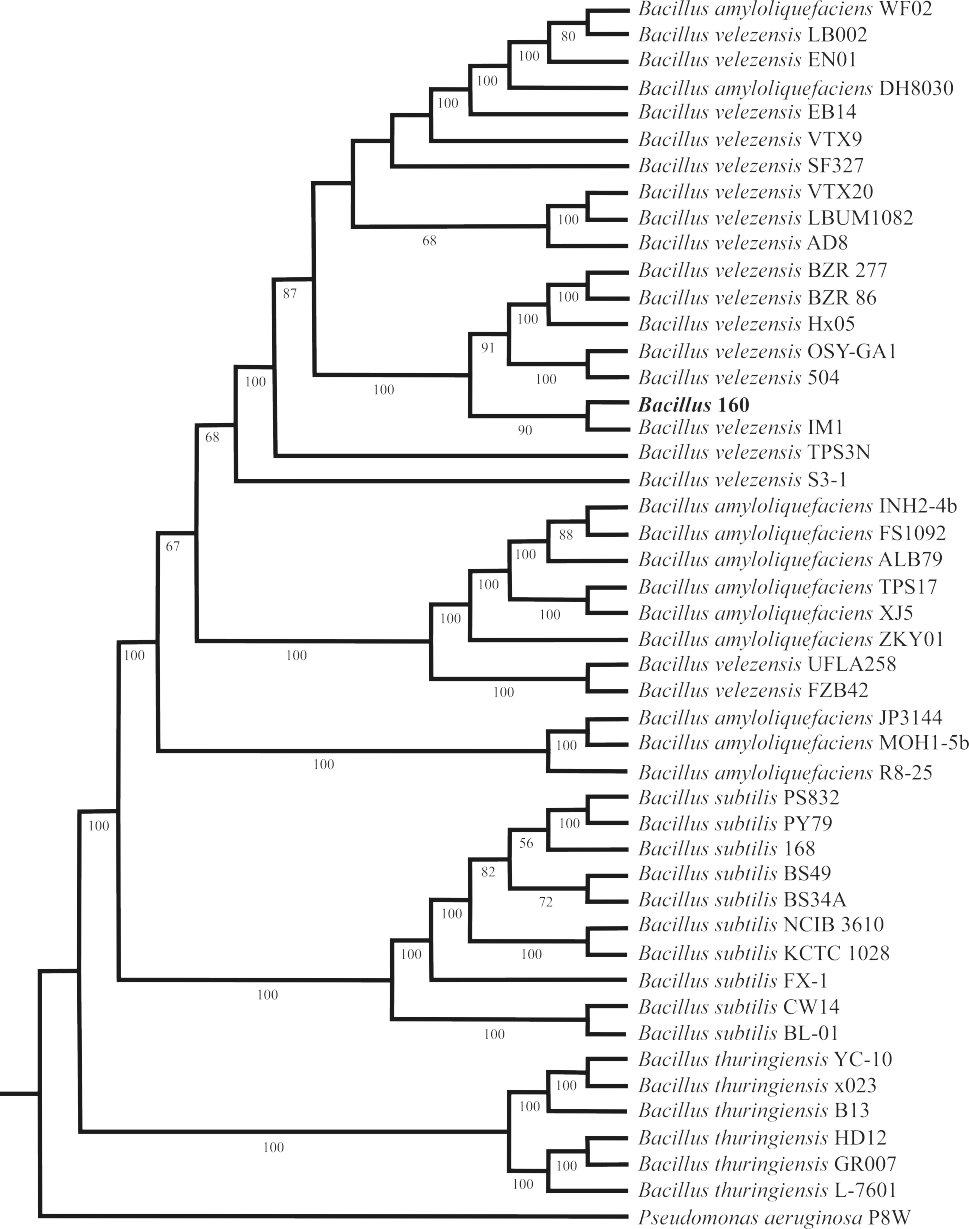
Phylogenomic relationship between the *B. velezensis* 160 strain and various species of the *Bacillus* genus. The strain under study is indicated in red. The access codes of the genome sequences used are as follows: *B. velezensis* Hx05 NZ_CP029473, *B. velezensis* 504 NZ_CP092439, *B. velezensis* OSY-GA1 NZ_CP031880, *B. velezensis* IM1 NZ_CP041784, *B. velezensis* LBUM1082 NZ_CP065790, *B. velezensis* VTX20 NZ_CP075054, *B. velezensis* EN01 NZ_CP053377, *B. velezensis* LB002 NZ_CP037417, *B. velezensis* VTX9 NZ_CP074685, *B. velezensis* AD8 NZ_CP072310, *B. velezensis* SF327 NZ_CP092383, *B. velezensis* TPS3N NZ_CP085283, *B. velezensis* EB14 NZ_CP065473, *B. velezensis* BZR 277 NZ_CP064845, *B. velezensis* R86 NZ_CP064846, *B. velezensis* S3-1 NZ_CP016371, *B. velezensis* UFLA258 NZ_CP039297, *B. velezensis* FZB42 NZ_CP000560, *B. amyloliquefaciens* FS1092 NZ_CP038028, *B. amyloliquefaciens* INH2-4b NZ_CP061852, *B. amyloliquefaciens* ALB79 NZ_CP029071, *B. amyloliquefaciens* TPS17 NZ_CP085282, *B. amyloliquefaciens* XJ5 NZ_CP071970, *B. amyloliquefaciens* ZKY01 NZ_CP044132, *B. amyloliquefaciens* MOH1-5b NZ_CP061853, *B. amyloliquefaciens* JP3144 NZ_CP082283, *B. amyloliquefaciens* R8-25 NZ_CP054479, *B. amyloliquefaciens* WF02 NZ_CP053376, *B. amyloliquefaciens* DH8030 NZ_CP041770, *B. subtilis* KCTC 1028 NZ_CP011115, *B. subtilis* NCIB 3610 , *B. subtilis* 168 NC_000964, *B. subtilis* BS49 NZ_LN649259, *B. subtilis* BS34A NZ_LN680001, *B. subtilis* PS832 NZ_CP010053, *B. subtilis* PY79 NC_022898, *B. subtilis* FX-1 NZ_CP061870, *B. subtilis* BL-01 NZ_CP028812, *B. subtilis* CW14 NZ_CP016767, *B. thuringiensis* YC-10 NZ_CP011349, *B. thuringiensis* x023 NZ_CP045585, *B. thuringiensis* B13 NZ_CP074714, *B. thuringiensis* HD12 NZ_CP014847, *B. thuringiensis* GR007 NZ_CP076539, *B. thuringiensis* L-7601, and *P. aeruginosa* P8W CP081477.2.

The sequencing process performed with the genome under study allowed us to obtain sequences of acceptable quality. We obtained 415 contigs from the genome assembly, of which five were eliminated because they were shorter than 250 bp, leaving 410 with an N50 value of 223,262. Low-complexity sequences shorter than 100 bp were also eliminated. The 410 contigs were joined to form one single 4,296,610 bp contig that constituted one chromosome. When this was compared with the contig of strain *Bacillus velezensis* EB14 using the QUAST software, a value of L50 of 1 was found, which allowed to corroborate the quality of the assembly. The sequence obtained was used to carry out the phylogenomic analysis, in which 46 sequences of the complete genomes of various *Bacillus* species were selected from the BLAST-N analyses, including *B. velezensis*, *B. amyloliquefaciens*, *B. subtilis*, and *B. thuringiensis*, as well as the *Pseudomonas aeruginosa* P8W genome as an outgroup. The phylogenomic tree constructed shows that the strain of interest was grouped with *B. velezensis* (Fig. 1). The genetic distances of strains of the clade of *B. velezensis* with respect to *B. velezensis* 160 were between 0.0658 and 0.0987, suggesting that they are the same species, with values lower than 1. In addition, the similarity of the genome sequences of the aforementioned *Bacillus* species with the strain under study was evaluated, finding an ANI (Average Nucleotide Identity) of 99%–100% similarity with *B. velezensis*.

### Genome features and functional annotation

**Fig 2:**
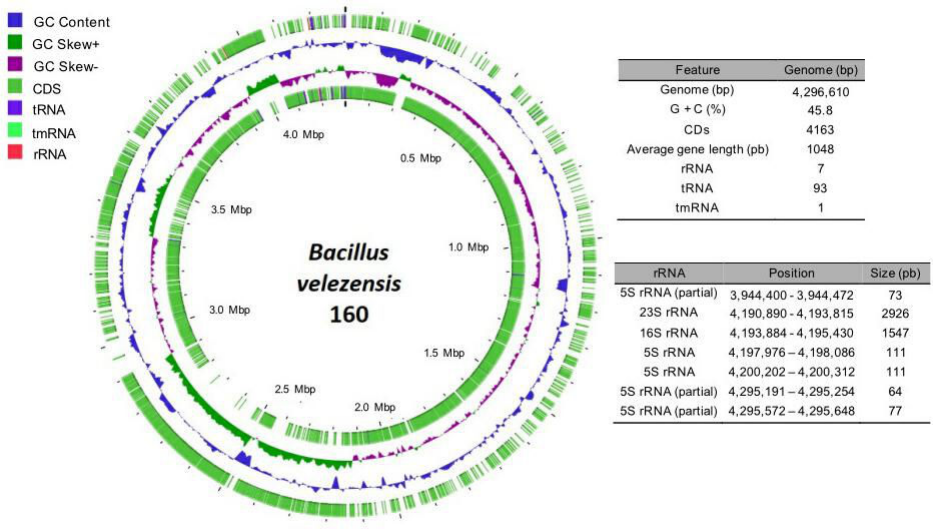
Genomic map of the chromosome of *B. velezensis* 160. The outer and innermost circles represent the location of the forward and reverse CDSs, respectively. The second and third circles from the outermost circle represent the GC content and GC skew, respectively. The characteristics of the chromosome and the position and size of the rRNAs are also shown in the tables.

The *B. velezensis* 160 genome comprises a single, circular chromosome with a length of 4,297,348 bp, a GC content of 45.8%, and 4,174 coding sequences (CDSs), representing 90.2% of the complete genome sequence. The chromosome contains seven genes of rRNA, of which an operon is formed that includes the 23s RNA and 16s RNA genes, as well as 93 tRNAs and 1 tmRNA. The orthologous genes present were 3,182. The gene sequences with unknown functions comprise 29% of the predictions (Fig. 2).

**Fig 3:**
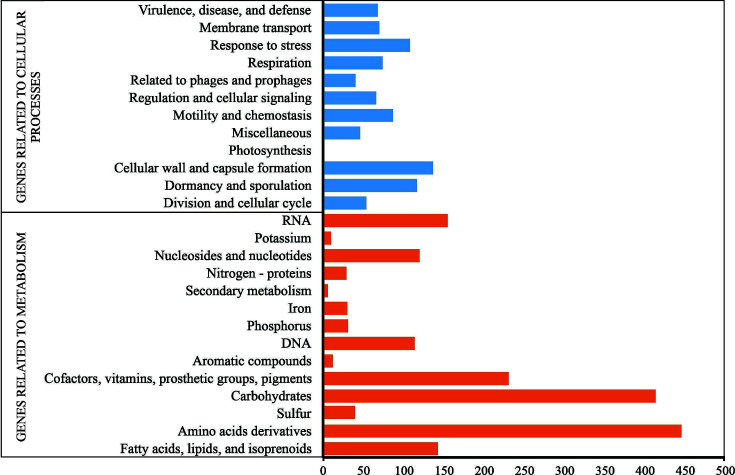
Predicted gene classification of the genome of *B. velezensis* 160, generated through annotation using the classic RAST software.

The genome annotation carried out by the RAST server allowed us to verify the classification of the predicted genes mainly in two categories: 868 and 1,781 related to cellular processes and metabolism, respectively. In the latter group, we observed that the genome presents a large number of genes related to carbohydrate metabolism, as well as to amino acids and their derivatives (Fig. 3).

### Comparative analysis

**Fig 4:**
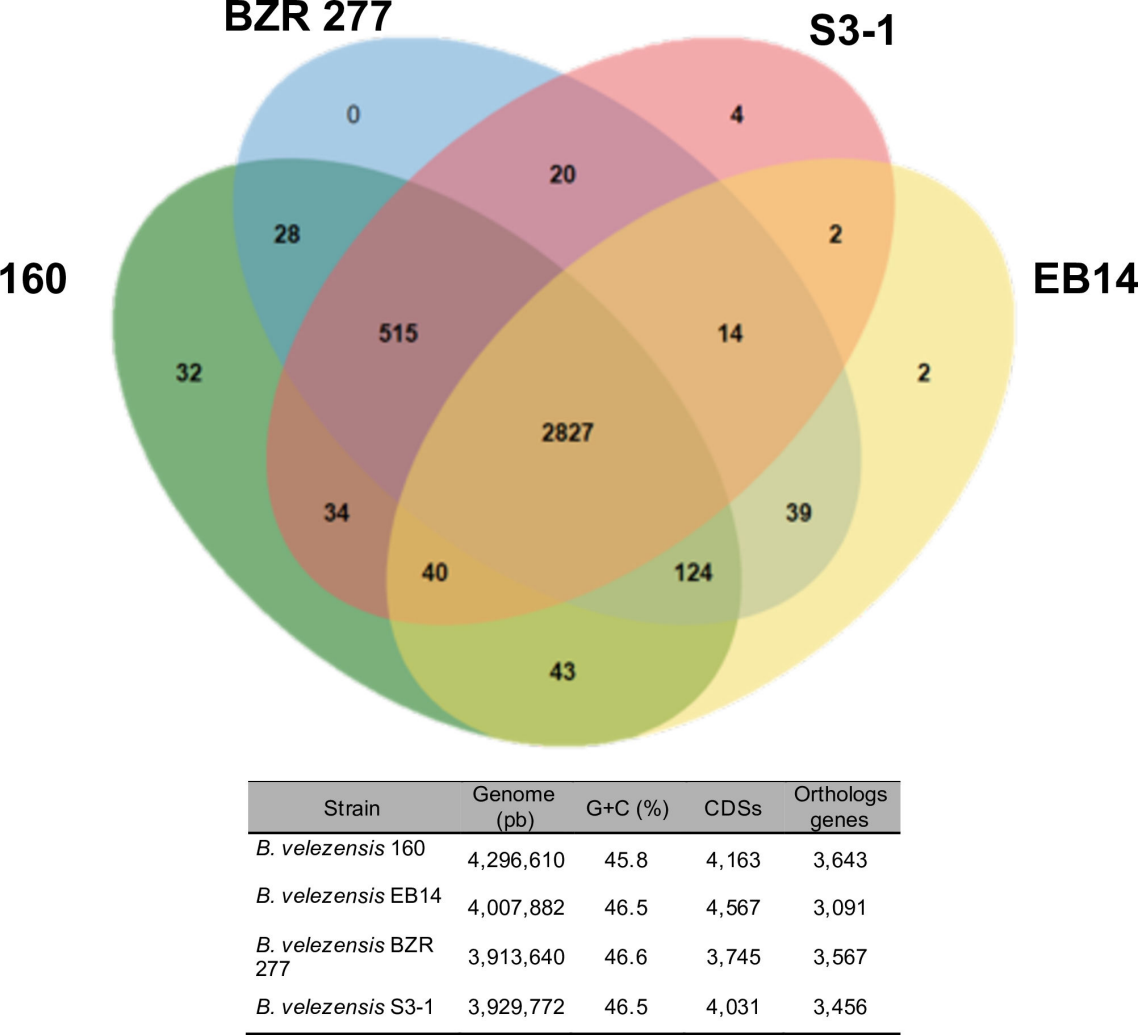
Comparative analysis of the genomes of several strains of *B. velezensis*. Representation in a Venn diagram and comparative table. The access codes of the genomes in GenBank are the following: *B. velezensis* 160 from this study, CP119675.1; *B. velezensis* EB14, CP065473.1; *B. velezensis* S3-1, CP016371.1; and *B. velezensis* BZR 277, CP064845.1. The number of coding regions is among the subsets of each genome.

For the comparative analysis, we selected the genomes of the following three strains of *B. velezensis* that were phylogenomically related to strain 160 and are used as biological control agents were selected: EB14, S3-1, and BZR 277. All these strains were found to share 2,804 genes, whereas *B. velezensis* 160 did not share 32 genes with the others. The strain of interest had the largest genome in terms of bp (Fig. 4).

### Genes related to the production of secondary metabolites with antimicrobial activity

The analysis performed using antiSMASH enabled us to identify the genetic potential of *B. velezensis* 160 to produce the following secondary metabolites with antimicrobial activity: laterocidin, difficidin, bacillibactin, bacilysin, macrolantin, bacillaene, fengycin, butirosin A, surfactin, and locillomycin, as well as three unknown compounds.

**Fig 5:**
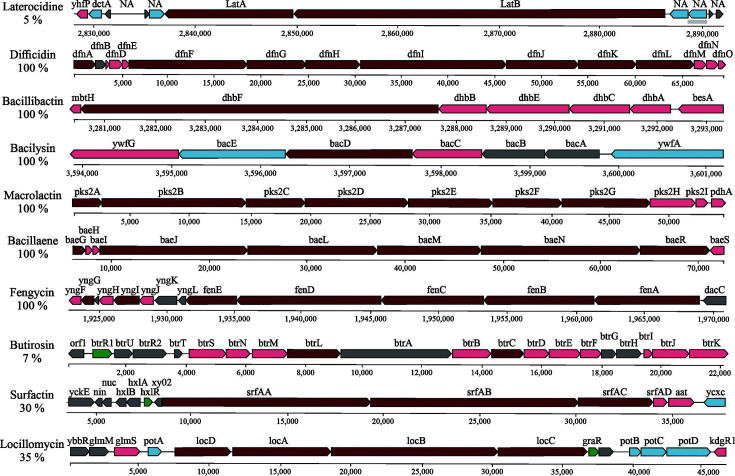
Graphic representation of the gene clusters related to the production of secondary metabolites of *B. velezensis* 160 with antimicrobial action. Numbers in gray indicate the position in the genome. ■, core biosynthetic genes; ■, additional biosynthetic genes; ■, transport-related genes; ■, regulatory genes; ■, other genes.

Figure 5 illustrates graphically the gene clusters related to the aforementioned metabolites and their position in the genome. All central genes are associated with the biosynthesis of lipopeptides (laterocidine, fengycin, surfactin, and locillomycin), macrolides (difficidin and bacilysin), one siderophore (bacilibactin), polyketides (macrolactin and bacillaene), and an aminoglycoside (butirosin A) (Table 1).

**TABLE 1:** Core biosynthetic genes for metabolite production with antimicrobial action identified in the *B. velezensis* 160 genome

Metabolite	Gene	Position
Laterocidine	*latA*	2837039–2849647
	*latB*	2849823–2886395
Difficidin	*difA*	1–2376
	*difF*	5847–18437
	*difG*	18456–24752
	*difH*	24765–30518
	*difI*	30570–46184
	*difJ*	46189–53907
	*difIK*	53930–60082
Bacillibactin	*dhbF*	3280519–3287655
Bacilysin	*bacD*	3596267–3597685
Macrolactin	*pks2A*	1–2307
	*pks2B*	2329–14589
	*pks2C*	14589–19361
	*pks2D*	19379–28117
	*pks2E*	28110–35114
	*pks2F*	35129–40849
	*pks2G*	40846–48228
Bacillaene	*baeG*	5581–6858
	*baeJ*	8427–23375
	*baeL*	23377–36804
	*baeM*	36822–47357
	*baeN*	47347–63648
	*baeR*	63662–71110
Fengycin	*fenE*	1931328–1935131
	*fenD*	1935150–1945925
	*fenC*	1945951–1953600
	*fenB*	1953616–1961313
	*fenA*	1961339–1968997
Butirosin A	*btrL*	7411–9225
	*btrC*	14291–15397
Surfactin	*srfAA*	8558–19312
	*srfAB*	19334–30094
	*srfAC*	30129–33965
Locillomycin	*locD*	7565–11446
	*locA*	11667–18644
	*locB*	18726–30332
	*locC*	30402–36782

**Fig 6:**
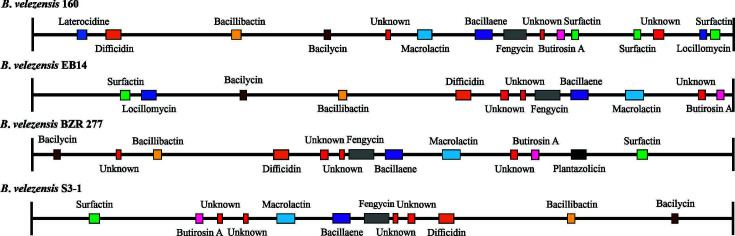
Comparison of clusters related to the production of secondary metabolites with antimicrobial activity in different strains of *B. velezensis*.

The comparison of the presence of clusters related to the production of antimicrobial metabolites of the strain of interest with the *B. velezensis* strains mentioned above revealed that *B. velezensis* 160 had three clusters for surfactin production, whereas the others had only one. Another difference is the potential of strain 160 to produce laterocidin, which is not found in the others. Unlike strains 160 and EB14, *B. velezensis* BZR 277 and S3-1 do not have clusters that code for the synthesis of locillomycin. All strains had clusters to produce difficidin, bacillibactin, bacilysin, macrolantin, bacillaene, butirosin A, surfactin, fengycin, and unknown compounds. Furthermore, strain BZR 277 also had clusters for producing plantazolicin (Fig. 6). In contrast, the analysis of *B. velezensis* 160 with PRISM 3 allowed the identification of 15 clusters for the synthesis of secondary metabolites with antimicrobial activity (2 for polyketides, 11 for non-ribosomal peptides, 1 for bacilysin, and 1 for a lanthipeptide).

## DISCUSSION

Some members of the genus *Bacillus* are widely known for their importance as biological control agents for plant pathogens, which has led to their increasing use in agriculture (26, 27). Advances in DNA sequencing technology have been essential for studying these organisms. The tools currently available make it possible to obtain the genome sequence of a bacterium within hours. In this regard, the genomic characterization of rhizobacteria has proven to be beneficial in providing greater insight into their potential uses and interactions with plants (28, 29).

As this study developed, we determined that the strain initially identified as *B. subtilis* 160 is actually *B. velezensis*. This bacterium is considered synonymous with *Bacillus amyloliquefaciens* subsp. *plantarum* or *Bacillus methylotrophicus*, a widely distributed PGPR in nature that in recent years, has gained attention for its ability to produce various metabolites with antimicrobial action and stimulate plant development (30, 31).

The antifungal capacity of *B. velezensis* 160 has been demonstrated by its ability to inhibit the development of *S. reilianum*, the causal agent of corn head smut. Applying it in field conditions reduced the incidence of this disease and increased crop productivity (25). It can also inhibit the *in vitro* development of the phytopathogenic fungi *S. maydis* and *S. macrospora*, which cause rotting in corn crops and are important producers of mycotoxins during storage (24).

The genome of *B. velezensis* 160 was compared to those of three other strains with antifungal activity: EB14, an endophytic bacterium isolated from poplar leaves (32); S3–1, obtained from the rhizosphere of cucumber crops (33); and BZR 277, obtained from the rhizosphere of rapeseed crops (34). All four share a significant number of genes; however, strain 160 presented the highest number of unshared coding sequences and was found to have the largest genome. A comparative genomics study of several *Alteromonas* sp. strains showed that the SN2 strain isolated from hydrocarbon-contaminated sea-tidal flat sediment had a larger genome than other strains of the same genus, which may confer ecological aptitudes that enable it to metabolize hydrocarbons in a habitat where temperatures fluctuate significantly throughout the year. Furthermore, it is prevalent in cold environments (35). As mentioned previously, *B. velezensis* 160 was isolated from the rhizosphere of corn crops sampled in the Mezquital Valley in Mexico (24). This valley is well known for harboring the largest agricultural area irrigated with sewage water (36), resulting in increased salinity and high levels of chemical and biological contamination in the soil (37, 38). The 160 strain analyzed here in likely has specific genetic components that allow it to thrive in such an ecosystem. This is demonstrated by its large genome size and the three clusters that code for the synthesis of surfactins, a characteristic genetic trait that could be linked to its effectiveness in biocontrol.

Fifteen clusters of genes related to the production of antimicrobial substances were identified in the genome of *B. velezensis* 160. Three are of unknown identity. Previous reports indicate that various strains of *B. velezensis* have the capacity to synthesize several non-ribosomal peptides, such as surfactins, bacillomycin D, fengycin, bacilibactin, ituirin, locillomycin, and bacillothiazol, besides polyketides (macrolactin, bacillaene, and difficidin), as well as other antimicrobials, such as bacilysin, butirosin, and plantazolicin (27, 39–42). Other reports have demonstrated the synthesis of some of the aforementioned antimicrobials. For example, the Y6 and F7 strains of *B. velezensis* have demonstrated antagonistic activity against the phytopathogens *Fusarium oxysporum* and *Ralstonia solanacearum* due to the production of surfactins, ituirins, and fengycins. In this case, the synthesis of lipopeptides is stimulated by the presence of *R. solanacearum* (43). The *B. velezensis* strain IP22 exhibits antimicrobial activity against *Xanthomonas euvesicatoria* due to its production of lipopeptides from the fengycin and locillomycin families (44). Another study showed that the *B. velezensis* FZB42 mutant in bacilysin biosynthesis loses its antifungal effect on the oomycete phytopathogen *Phytophthora sojae* (45), whereas the *B. velezensis* LM2303 strain exhibits a strong antifungal effect on *Fusarium graminearum* due to its capacity to produce fengycin B, iturin A, surfactin A, butirosin, plantazolicin, kijanimicin, bacilysin, difficidin, bacillaene A, bacillaene B, and macrolactin A (46). The analysis of the genome of the various strains of *B. velezensis* offers a wide scope of study that allows a connection to be drawn between genetic information and *in vivo* behavior.

A cluster for producing laterocidine was identified in *B. velezensis* 160, which has been reported as a secondary metabolite of *Brevibacillus laterosporus*. This antimicrobial was evaluated against the ESKAPE pathogens (*Enterococcus faecium*, *Staphylococcus aureus*, *Klebsiella pneumoniae*, *Acinetobacter baumannii*, *Pseudomonas aeruginosa*, and *Enterobacter* species) and was found to inhibit Gram-negative bacteria. Thus, it could be a viable option for combating multi-resistant bacteria (47). The *B. velezensis* 160 strain may have acquired this genetic information through horizontal gene transfer, a process that has been proposed as an essential mechanism in adapting rhizosphere bacteria that could be related to their colonizing ability (48).

The results of this study will enable further characterization of strain 160, now *B. velezensis*, a biological control agent for corn ear smut. It has been established that genome sequencing can be used to identify and classify microorganisms and explore their metabolic properties (49). Therefore, this approach can contribute to the characterization of rhizobacteria that could increase crop productivity and benefit agriculture.

## MATERIALS AND METHODS

### Microorganism and conservation

The bacterial strain identified as *B. subtilis* 160, used in this work, was isolated from the rhizospheric soil of maize crops in the community of Cinta Larga, municipality of Mixquiahuala Hgo, state of Hidalgo, in central Mexico (24). The strain was stored in cryotubes with 25% glycerol at −70°C.

### Extraction and genomic DNA sequencing

For the extraction of genomic DNA, the contents of a cryotube with the strain were inoculated into 50 mL of Luria Bertani broth and then incubated at 28°C at 150 rpm for 48 h. An aliquot was taken from this culture to adjust 5 mL of the same medium to 0.2 absorbance at 600 nm, followed by incubation for 24 h under the same conditions. Finally, all the biomass was collected in an Eppendorf tube by centrifugation, from which the genomic DNA was extracted by the cetyltrimethylammonium bromide method (50). DNA integrity was confirmed through electrophoresis in 1% agarose gel. DNA quality and quantity were then determined using a NanoDrop spectrophotometer. Sequencing was carried out using the Novogene Co.’s service on an Illumina platform (paired-end readouts of 150 bp length). DNA library preparation was conducted using the NEBNext Ultra II DNA Library Prep Kit for Illumina.

### Data quality control and genome assembly

The raw data were analyzed for quality control using the FastQC software version 0.11.5 (51). Subsequently, Trimmomatic version 0.32 (52) removed the adapters and low-quality sequences (<250 bp). Finally, *de novo* assembly was performed using Spades version 3.15.4 (53), considering K-MERS of 1,000, 5,000, 10,000, 25,000, and 50,000 bp. This same software determined the L50 and N50 values. To verify the assembly quality, the complete genome of *B. velezensis* 160 was compared with the genome of *B. velezensis* EB14 (Access Code NZ_CP085283) with the QUAST software (54).

### Phylogenomic analysis

Once the quality analysis of the genome was approved, the sequence obtained was compared to the genomes deposited in the GenBank Database of the National Center for Biotechnology Information (NCBI) using the BLAST-N software (https://blast.ncbi.nlm.nih.gov/Blast.cgi). Based on the results obtained, genome sequences of different *Bacillus* species with similarity values of ≥98% were selected and used to create a phylogenomic tree using the software M1CR0B1AL1Z3R (55) and itol (https://itol.embl.de). A maximal *e*-value cutoff of 0.05, a minimal percent cutoff of 20%, and a minimal percentage for the core of 100.0% were considered. In addition, the genetic distances between the strains of the *B. velezensis* clade were calculated using the online software DSMZ (56). The ANI tool (Average Nucleotide Identity: http://enve-omics.ce.gatech.edu/ani/) was used to estimate the similarity of the genomic sequences of the different *Bacillus* species used in this study (57).

### Functional annotation and identification of secondary metabolites

The functional annotation of the genome of *B. velezensis* 160 was carried out using the Prokka version 1.14.6 (58) and RAST (59) software. The ideogram was constructed utilizing the CGview server (60) with the results from Prokka. The identification of secondary metabolites in genome mining was carried out using the antiSMASH software version 6.0.0. and PRISM 3 (61, 62).

### Comparative analysis

The genome of strain 160 was compared to genomic sequences of other *B. velezensis* strains considered as rhizobacteria and used as biological control agents obtained from NCBI GenBank (https://www.ncbi.nlm.nih.gov/genome/). This analysis was carried out using the Orthovenn2 software (63).

## Data Availability

The genome sequence was deposited in the GenBank database under BioProject number PRJNA938964 and accession number CP119675.1.
